# Beyond Imaging: Localized Synthesis, Fabrication, and Surface Modification via Scanning Electrochemical Cell Microscopy

**DOI:** 10.1021/acsnano.6c03778

**Published:** 2026-08-26

**Authors:** Bethanie Dean, Patrick Beard, Anna Pushkareva Fazullina, Dimitrios Valavanis, Noah Al-Shamery, Joshua Magiera, Jordan S. Lee, Pooi See Lee, Patrick R. Unwin, Paul Wilson

**Affiliations:** † Department of Chemistry, 2707 University of Warwick, Coventry CV4 7AL, U.K.; ‡ Analytical Science CDT, University of Warwick, Coventry CV4 7AL, U.K.; § School of Materials Science and Engineering, 54761 Nanyang Technological University, 50 Nanyang Avenue, Singapore 639798, Singapore; ∥ Hartnoll Centre for Experimental Fuel Technologies, University of Warwick, Coventry CV4 7AL, U.K.

**Keywords:** SECCM, localized electrochemistry, nanoscale synthesis, crystallization, nucleation, surface transformation, site-selective deposition, meniscus-confined reactions

## Abstract

Meniscus-confined electrochemical deposition (MCED) enables highly localized material synthesis, transformation, and surface modification. The Perspective highlights scanning electrochemical cell microscopy (SECCM) as a highly versatile MCED platform. While primarily developed for electrochemical imaging, SECCM also functions as a direct-write tool capable of producing complex architectures and probing associated localized electrochemical phenomena. We focus on a concise technical overview to position SECCM within a broader MCED landscape. We then examine its application across key material processes: metal and metal alloy deposition, surface functionalization and reagent delivery, and polymer synthesis. In each case, SECCM not only enables material fabrication or dissolution with high spatiotemporal control but is also capable of revealing nanoscale mechanistic insights from the analysis of the electrochemical response and/or the use of integrated capability, such as complementary microscopy. By framing SECCM as a fabrication platform, this work aims to connect the SECCM and MCED communities and encourage interdisciplinary adoption. We discuss emerging opportunities in device integration, materials design, and fundamental electrochemistry as well as key practical challenges while outlining future directions for expanding the capability of SECCM as a nanoscale manufacturing tool.

## Graphical abstract

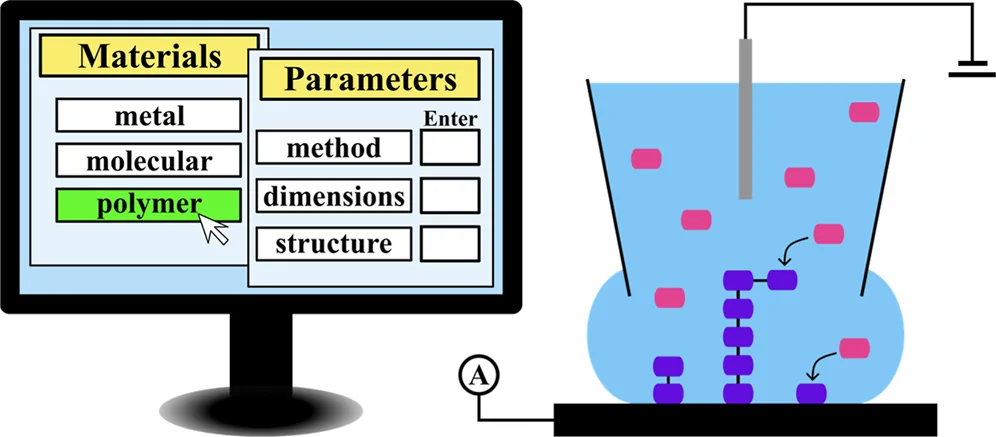

## 1 Introduction

Precise spatiotemporal control over electrochemical reactions is becoming increasingly important for designing next-generation materials and devices, with applications in catalysis,^1,2^ nanoelectronics,^3^ and surface engineering.^4^ Electrochemical methods offer a direct and scalable route for material deposition,^5^ transformation,^6^ and removal,^7^ including controlled crystallization,^8^ patterned polymer growth,^9^ and site-selective functionalization.^10^ Unlike conventional top-down fabrication approaches such as photolithography or focused ion beam (FIB) techniques,^11,12^ maskless additive manufacturing with real-time tunability of conditions can be accessed through an applied potential or current.^13^ Additionally, these systems may also be operated under ambient or readily controlled conditions,^14^ avoiding harsh environments required by high-energy radiation or ultrahigh vacuum processes.^15^

To facilitate local patterning and surface modification, a family of techniques have been developed that use a liquid meniscus to spatially confine electrochemical reactions to a defined contact zone.^16^ Collectively referred to in this Perspective as meniscus-confined electrochemical deposition (MCED), these approaches occupy a unique intersection between electrochemistry, scanning probe microscopy (SPM), and submicrometer manufacturing.^17–19^

Among these, fluidic force microscopy (FluidFM) has received considerable attention, providing a well-established example of MCED in the literature.^20–23^ By combining the accurate positioning of atomic force microscopy (AFM) with fluidics delivered by a microchannel within the AFM cantilever, FluidFM enables local liquid dispensing at submicrometer apertures. While initially intended for single-cell manipulation,^24^ research has demonstrated the power of FluidFM in the manufacture of complex and sophisticated microstructures via electrodeposition. One of the most prominent examples of this is a 1:70,000 replica of Michelangelo’s David.^25^ Although not the focus of this Perspective, FluidFM has been extensively covered in several recent focused reviews.^26–28^

FluidFM can be operated in single phase (where the sample is immersed in a bath solution similar to that in the tip) or dual phase (where (i) immiscible liquids are in the tip and bath or (ii) liquid is in the tip and the sample is in a gaseous or air environment). Scanning ion conductance microscopy (SICM)—a related SPM technique to FluidFM—operates by the immersion of the sample into a bath of electrolyte solution but differs by the use of a glass micro- or nanopipet with defined geometry in place of a cantilever. Ion transport between a quasi-reference counter-electrode (QRCE) in the pipet and a QRCE in the bath solution is modulated by the nearby sample surface, affecting the current response. Similar to FluidFM, SICM can simultaneously map surface topography and measure or modify the properties of a substrate surface.^29,30^ SICM-based MCED has found applications in metal nanostructure fabrication, including the manufacture of freestanding copper microwires.^31^

Scanning electrochemical cell microscopy (SECCM) may employ similar single-channel glass pipets as typically used in SICM, but multichannel probes are also extensively used. The pipet itself is used as the electrolyte solution reservoir.^18,32^ The liquid meniscus formed at the pipet tip establishes contact with the surface under study (without the probe touching the surface), and the local cell formed can be used to promote local electrochemical reactions.^33,34^ In the simplest configuration, a QRCE inserted into the pipet and a conductive substrate (typically, although insulating substrates can also be used) complete the two-electrode system, and a potential can be applied between these to drive a reaction.^18,35^ SECCM and closely related techniques have been used for the fabrication of a range of materials, from metallic nanoparticles^36^ to microstructures,^37^ to polymeric towers^38^ and biomaterials.^39^

While all of the techniques discussed above offer submicrometer additive manufacturing with *operando* topography measurements and fast data acquisition,^40^ each technique naturally has inherent limitations and challenges. Appropriate FluidFM cantilevers are expensive and difficult to manufacture.^41,42^ Despite resolution capabilities of 1 nm in the *z*-axis, the highest *x–y* resolutions are typically on the 200 nm scale, governed by the tip apertures, which are presently difficult to manufacture on a finer scale.

SICM- and SECCM-based MCED do not suffer from the same tip aperture limitations. Glass pipets are easy to fabricate using commercial pipet pullers (e.g., thermal or CO_2_ laser pullers) and are relatively cheap in comparison to cantilevers for FluidFM. Pipet tip apertures can be readily modified between ∼10 μm and ∼10 nm from user-defined programs.^43^ This range uniquely offers experimental scalability between ultrahigh resolution to access mechanistic insights at the single particle level and ensemble-scale measurements to enable the construction of complex structures and interfaces. The ability to study systems with a multiscale understanding, owing to a readily tuned probe size, is a particular advantage of pipet-based probe systems.

Although SICM offers high spatial resolution, there is some broadening of the resolution due to diffusion of the electrolyte from the tip into the bath.^44^ For SECCM, the meniscus size is the predominant factor that determines spatial resolution. While the liquid meniscus at the end of a SECCM tip has been characterized to a high level, and is stable under a wide range of conditions,^8,45^ it is useful to be mindful of evaporation effects. These effects may drive local concentration gradients and cause supersaturation at the outer edges of the meniscus–substrate boundary (especially when using volatile solvents or aqueous solution at a low relative humidity), leading to the well-known “coffee ring” effect.^46^

Advances pertaining to the use of SECCM as a characterization tool, including practical guidance and technical capabilities, have been comprehensively reviewed elsewhere.^32,47–53^ The Perspective is distinct in focusing on SECCM as a synthetic manufacturing platform while retaining the high-resolution, high-throughput, and customizability traits that underscore its broad usage. In this Perspective, we consider the fabrication of metal nanomaterials from nucleation to 3D nanostructures, molecular grafting and deposition, and finally polymeric systems to illustrate the versatility of SECCM and meniscus-guided approaches in studied systems of increasing complexity.

SECCM is often absent from MCED conversations. One barrier to the broader engagement of SECCM in the MCED community is inconsistent terminology. Functionally similar approaches are described using a wide variety of names, including (i) electrochemical fountain pen nanofabrication,^54^ (ii) electrochemical dip-pen nanolithography,^55,56^ (iii) meniscus-guided electrochemical deposition,^57^ (iv) high-resolution three-dimensional (3D) printing,^58^ (v) meniscus-confined electroplating,^25^ (vi) electrodeposition-based 3D printing,^59^ and (vii) 3D μ-printing.^60^ These distinctions often reflect differences in instrumentation, research community or application area. By discussing SECCM as a representative and evolving member of the MCED family, we highlight the conceptual coherence across experimental approaches. The relative merits and challenges between approaches are compared to establish a unifying framework that will support broader accessibility, integration, and innovation in electrochemistry, materials science, and nanofabrication.

## 2 Overview of Scanning Electrochemical Cell Microscopy

In the single-barrel SECCM configuration, initially termed the scanning micropipet contact method,^35^ an electrochemical cell is established between a QRCE, inserted into the glass or quartz pipet filled with the electrolyte solution of choice, and a working electrode surface (Figure 1a).^52^ By biasing the QRCE potential versus the surface (indicated with *V*_1_ in Figure 1a), a current (*i*_sub_) is generated upon meniscus–surface contact which can be used for positioning. It is noted that, owing to high-speed data acquisition and instrument control, the pipet probe itself does not make physical contact with the surface. Typically operated in a hopping-mode protocol (although other modes are available),^18,61^ the meniscus is landed at a predefined spot, a potential is applied to drive the reaction of interest, and the probe is retracted, breaking contact, and moved to the next predefined spot.^62^

**Figure 1:**
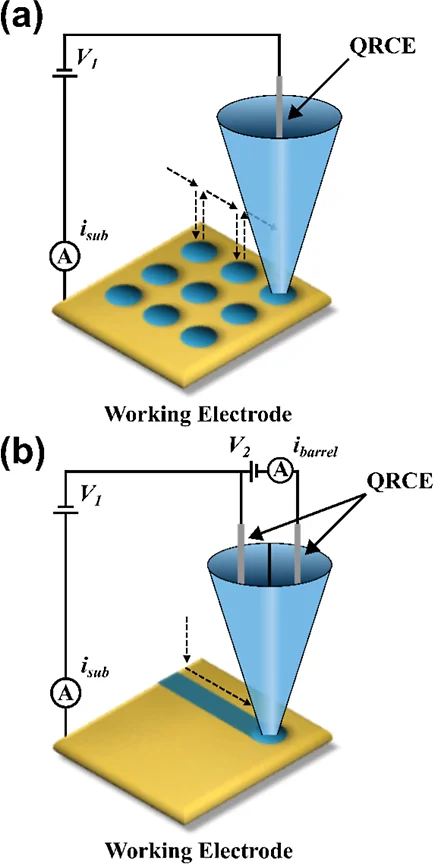
Schematics of two scanning electrochemical cell microscopy (SECCM) configurations. (a) Single-barrel pipet filled with electrolyte solution and an appropriate quasi-reference counter-electrode (QRCE). A potential (*V*_*1*_) is applied with respect to the substrate (i.e., working electrode; WE) where the current (*i*_*sur*f_) is measured. A hopping-mode protocol is depicted, where meniscus–surface contact is made at individual locations with retraction of the pipet between measurements. (b) Dual-barrel (*theta*) pipet has one QRCE in each of its two half-cone compartments. An additional potential is applied between the barrels (*V*_*2*_) to provide an ionic current (*i*_*barrel*_) to be used as positional feedback independent from *i*_*surf*_. A constant contact regime is depicted, where a defined meniscus–surface distance is maintained while the pipet moves laterally without retraction of the pipet from the surface. Note that all schematics are generated by authors and are not to scale.

The dual-barrel (or *theta*) pipet variants contain a solution and a QRCE in each of the two half-cone pipet compartments or barrels (Figure 1b).^18,61^ A bias applied between the QRCEs (indicated with *V*_2_ in Figure 1b) induces an ionic current through the meniscus, with changes in its geometry clearly reflected in the recorded current. This allows for the meniscus–surface contact to be detected with high reproducibility on any substrate material, irrespective of its conductivity. The sensitivity can be enhanced further, and the application field widened, via modulating the probe normal to the surface and comparing the modulation and response signals with a lock-in amplifier. This configuration enables active positional feedback to maintain a constant pipet–surface distance during lateral movement and facilitates precise planar patterning and etching without meniscus detachment. Conductive substrates can serve as the working electrode, and an additional independently set potential (*V*_1_) can drive surface electrochemical reactions without obstructing the active positional feedback mechanism.

The spatial resolution of SECCM is defined by the meniscus contact area, which depends on a combination of pipet geometry, electrochemical parameters (such as potential scan/sweep rate), surface properties (such as electronegativity), and environmental factors (such as temperature and humidity).^53,63^ Smaller pipet apertures produce smaller menisci, restricting the reaction area and allowing for an improved lateral resolution. While typical pipet diameters range from several micrometers to tens of nanometers,^52^ single-barrel pipets have achieved reported apertures of less than 10 nm in diameter.^37^ Experiment success also depends to some extent on pipet approach and retraction dynamics, which must be explored to optimize results.

## 3 Metal Deposition

### 3.1 Metal Nucleation and Growth

Metal nucleation and growth processes are important for nanofabrication and the creation of nanostructures with diverse applications in catalysis and energy storage. The study of metal nucleation on small length and time scales can also answer fundamental questions about the physicochemical phenomena that govern those processes.

Microscopic methods, that confine reactions to droplet cells^64–66^ —in femtoliter to picoliter electrolyte volumes—supplied by a much larger reservoir, enable the survey of a large parameter space at a high throughput. As the droplet cell is moved from point to point across an electrode substrate, parameters such as potential, current, measurement direction, etc. can be varied; thousands of such measurements can be made in an experimental run. As a consequence, SECCM is increasingly being used for studies in this area, facilitating the detection and monitoring of distinct growing phases at the single entity level. By aid of ex situ and in situ characterizations,^67^ the deposits can be described fully and any substrate effect highlighted clearly. This workflow follows naturally from extensive works undertaken in imaging polycrystalline, and more generally heterogeneous surfaces, using SECCM.^68–70^ A statistically relevant sample of measurements can be realized at each type of substrate (e.g., crystallographic facet, defect concentration, pretreatment parameters, etc.).^71^ Furthermore, seemingly homogeneous substrates may feature higher nucleation-active sites that are only accessible by a microscale or nanoscale approach. Lastly, the confined aspect of the experiment may give rise to reaction pathways that demand a volume-limited or reactant-limited regime to progress.^72,73^

The electrodeposition of Pt nanoparticles was thoroughly explored in a work by Ornelas et al. by leveraging SECCM and transmission electron microscopy (TEM) for atomic-scale analysis of the deposits.^74^ Pt was deposited, from the solution in the pipet probe, onto carbon-coated TEM grid supports, by chronoamperometry at a range of overpotentials, allowing for colocated high-resolution structure characterization and evaluation of the deposition conditions. A variety of structures were identified post-experiment—amorphous, monocrystalline, and polycrystalline Pt nanoparticles (and aggregates thereof)—supporting a nucleation–aggregation growth^75^ and corroborating early reports on Pd nanoscale electrodeposition.^76^

Expanding on previous work that employed SECCM to investigate Ag electrodeposition on highly oriented pyrolytic graphite (HOPG),^77^ researchers in a recent study managed to probe site-specific Ag nucleation kinetics.^78^ SECCM voltammetric experiments revealed a different behavior between the basal plane and step edges of HOPG, while a stochastic model was developed to extract the nucleation rate and energetics. Here, the spatially confined electrode active area allowed for the recording of individual events and the construction of a map of the required nucleation overpotential.

Ag nucleation has been examined in two studies by the Hill research group with a thin coating of indium tin oxide (ITO) or C on a glass (or quartz) support as the electrode substrate of choice. First, SECCM was utilized as a precise nanoscale fabrication tool, with dynamic monitoring of the charge collected at the electrode, to yield ordered nanoparticles of individually controlled size (Figure 2a).^79^ This approach revealed patterns in the relationship between the nucleation rate and the applied potential bias, in turn, offering a descriptor for the effect of surface heterogeneity. The semitransparent electrode offers direct visualization of the deposited patterns via in situ optical imaging, enabling immediate inspection of the results. More recently, single Ag particle nucleation experiments were presented against quasi-equilibrium and time-dependent kinetic models.^80^ The latter was found appropriate in the analysis of the extensive data set acquired with SECCM and was capable of providing meaningful chemical quantities such as surface energies and kinetic rate constants.

**Figure 2:**
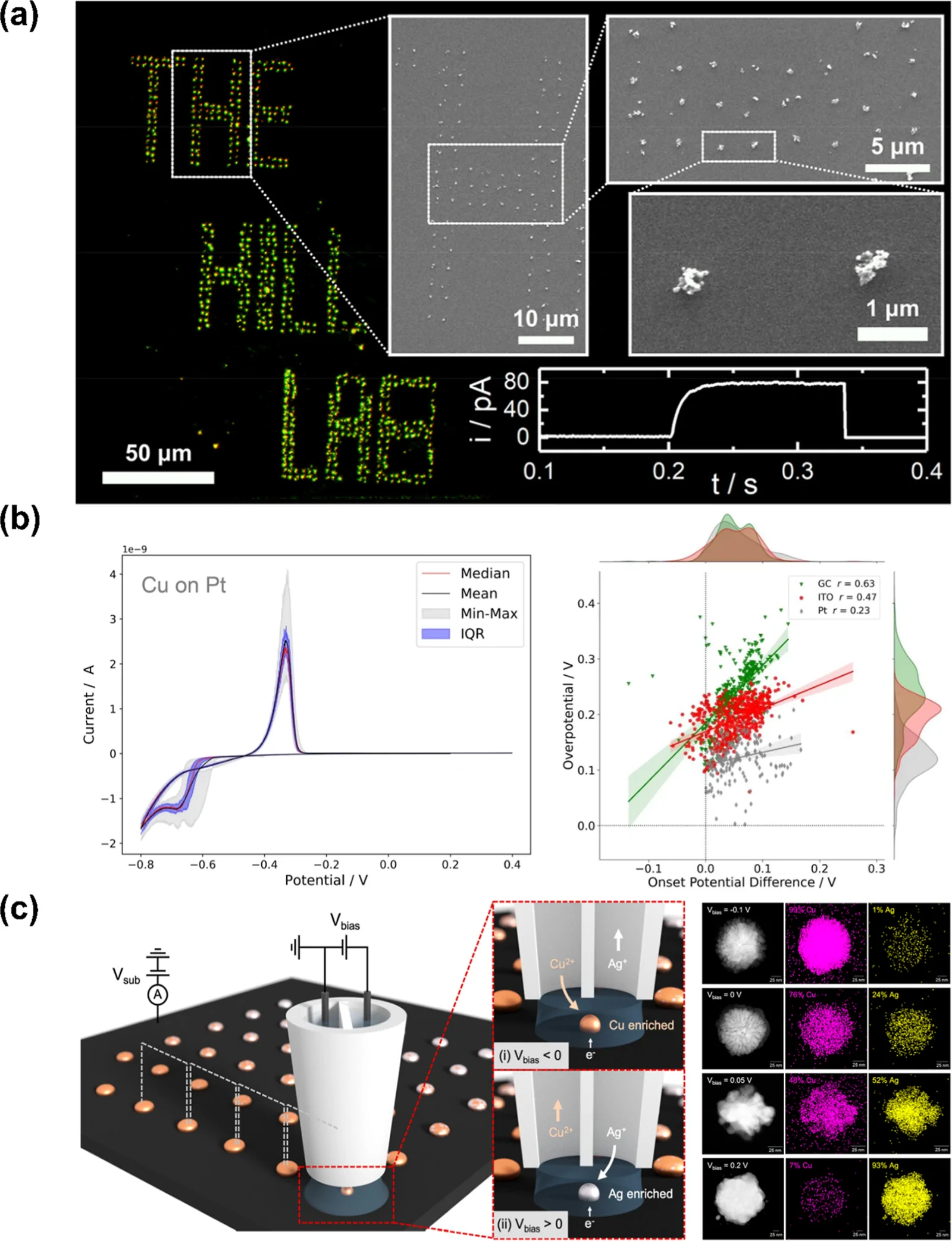
(a) Generating complex Ag nanoparticle patterns via scanning electrochemical cell microscopy (SECCM). Optical dark-field scattering image of electrodeposited Ag pattern on indium tin oxide (ITO) substrate (left) and scanning electron microscopy (SEM) images of a section of the particles (inset, top right). An exemplary current–time trace of an individual electrodeposition spot is also shown (bottom right). Reprinted with permission from Rahman, Md. M. et al. On-Demand Electrochemical Fabrication of Ordered Nanoparticle Arrays Using Scanning Electrochemical Cell Microscopy. ACS Nano **2022**, 16 (12), 21275–21282 (ref 79). Copyright 2022 American Chemical Society. (b) Unraveling the effect of surface state on metal nucleation through a cross-system examination. A summary of individual microscale Cu reduction and dissolution voltammograms on a Pt substrate, along with statistical quantities (left). Scatter plots of the overpotential of Cu electrodeposition versus the onset potential difference, comparing glassy carbon, ITO, and Pt as substrates. Legend indicates correlation coefficient quantified by r-square. Reprinted in part with permission from Torres, D. et al. Electrochemical Nucleation and the Role of the Surface State: Unraveling Activity Distributions with a Cross-System Examination and a Local Electrochemistry Approach. J Solid State Electrochem **2024**, 28 (5), 1719–1734 (ref 85). Copyright 2024 Springer Nature. (c) On-demand nanofluidic mixing for the electrodeposition of metal alloy nanoparticles. Schematic showing the controlled enriching, by the applied bias, *V*_bias_, from two reagents—Cu and Ag—from separate compartments of a dual-barrel SECCM pipet, into the nanoscale cell forming at the tip (left). Annular dark-field scanning TEM (ADF-STEM) images (first column) and the respective energy-dispersive X-ray spectroscopy elemental maps of Cu (middle column) and Ag (right column) from exemplary electrodeposited alloy nanoparticles, at chosen compositions (right). Adapted with permission from Lee, H. et al. Precision Synthesis of Bimetallic Nanoparticles via Nanofluidics in Nanopipets. ACS Nano **2023**, 17 (22), 22499–22507 (ref 86). Copyright 2023 American Chemical Society.

SECCM has been further used as a patterning tool to produce well-defined microscale spots of Ag nanoparticles in a study examining the antimicrobial effect of Ag against *Escherichia coli* bacteria.^81^ The technique was employed to screen electrodeposition parameters for Ag on polydopamine-coated Au substrates and then to construct an array of spots to be assessed for antimicrobial activity. Complementary AFM-based force spectroscopy revealed the bacterial outer membrane to undergo notable structural changes in close proximity to the Ag nanoparticles, an effect rationalized by multiphysics numerical simulations of Ag dissolution.

The electrochemical nucleation of Cu via SECCM is the focus of two related pieces of work that bring out the heterogeneity of glassy carbon (GC) electrode substrates at the microscale. By mapping stochastic nucleation events and leveraging a suite of characterization techniques (AFM; scanning electron microscopy, SEM; X-ray photoelectron spectroscopy, XPS), nucleation has been shown to be preferentially occurring at local features which are products of standard surface treatments, e.g., mechanical polishing or anodization.^82^ The collection of a large data set of localized measurements and the determination of descriptors support a thorough statistical analysis and the interpretation of nonuniform distribution of electrochemical activity (*electrochemical diversity*). In a follow-up study, the authors adapted a classical analytical model for describing current transients related to electrochemical nucleation and growth to consider the SECCM droplet cell geometry.^83^ Exploring different probe tip aperture (and in effect, electrode active area) sizes and overpotentials, they achieved correlation between current signatures and physical properties of the deposited nanoparticles.

The nucleation, growth, and dissolution of Au has been explored at the microscale by employing an array of SECCM voltammetric experiments on GC.^84^ A diversity of nucleation events at this scale—owing to electrode site-specific activity—was evaluated and contrasted with the inherently averaged response of macroscopic experiments. The work also provides insight into the dissolution of the previously electrodeposited NPs, highlighting stochastic current spikes during the anodic section of the voltammogram. This work was followed by a study on how surface state heterogeneities influence electrochemical nucleation activity distributions, demonstrating hundreds of localized measurements for the deposition of Cu or Ag on ITO, Pt, or GC substrates (Figure 2b).^85^ Cross-system analysis demonstrated that nucleation was not uniform but governed by localized surface states, a principle that questions the application of classical nucleation models. By investigating high- and low-energy substrates, these experiments revealed new aspects on electrochemical nucleation and growth processes that cannot be observed through macroscale experiments.

Fine-tuning of the electrodeposited particle properties is also an area of focus for SECCM studies, owing to the technique’s capacity for a limited reaction volume and a short parameter optimization cycle. The synthesis conditions of Pt nanoparticles were screened by SECCM and pulse potential electrode position to achieve crystallographic facet selectivity.^36^ The formation of (100) facets was found to be sensitive to the upper and lower potential limits of the pulse, while the density and size of the nanoparticles were also controlled. In a single experiment (scan) and subsequent electron microscopy session, multiple experimental parameters were evaluated, demonstrating the technique’s high-throughput capabilities.

A dual-barrel SECCM pipet can be used to synthesize bimetallic nanoparticles with highly controlled composition (Figure 2c).^86^ The working principle involves loading each pipet half-cone compartment with a different precursor solution and enriching on demand the SECCM meniscus with the metal ions of choice via an applied bias between the two barrels. Various metal combinations were explored (Cu–Ag, Cu–Pt, Au–Pt, Cu–Pb, and Co–Ni), and successful alloy production was confirmed by the analysis of anodic stripping and by the EDS elemental maps of the deposits.

The effectiveness of this approach was further illustrated in a follow-up work, where researchers regulated the spatial elemental distribution within the produced bimetallic nanoparticle structure.^87^ With fine control over the chemical composition of the SECCM droplet cell, along with the duration of the electrodeposition experiment, the synthesis of multilayer core–shell nanoparticles with tunable thickness, number, and sequence of layers becomes possible. Alloys of Pt–Cu and Pt–Ni were fabricated and then screened for their catalytic activity in hydrogen evolution and oxygen reduction reactions. Meniscus-guided bimetallic deposition via SECCM requires additional experimental factors to be considered. Mass transport from each solution in opposing pipet compartments, to and from the meniscus, needs to be resolved in detail, while there may be some distortion of the meniscus geometry when switching between different deposition conditions.^86^

In recent instrumentation advancements, SECCM has been coupled with in situ interference-based optical microscopy (interference reflection microscopy, IRM) to provide optical monitoring of the whole meniscus-wetted electrode active area synchronous to the electrochemical experiment. This is permitted by the use of the semitransparent and conductive substrates mentioned earlier, such as glass coated by a thin film of ITO, C, or Au. Showcasing the advantages of this hybrid approach, Godeffroy et al. carried out a multimicroscopy strategy, combining SECCM with in situ IRM and ex situ SEM, to characterize pathways in Ni electrodeposition (Figure 3a).^88^ They managed to distinguish between competing reaction routes, resulting in Ni or Ni­(OH)_2_ nanoparticles, each showing a different optical intensity profile: particles assigned to Ni appearing as bright-contrasted relative to the background intensity and measured (by SEM) to be relatively larger and spherical; and particles assigned to Ni­(OH)_2_ appearing dark-contrasted and measured to be relatively smaller and of fractal shape.

**Figure 3:**
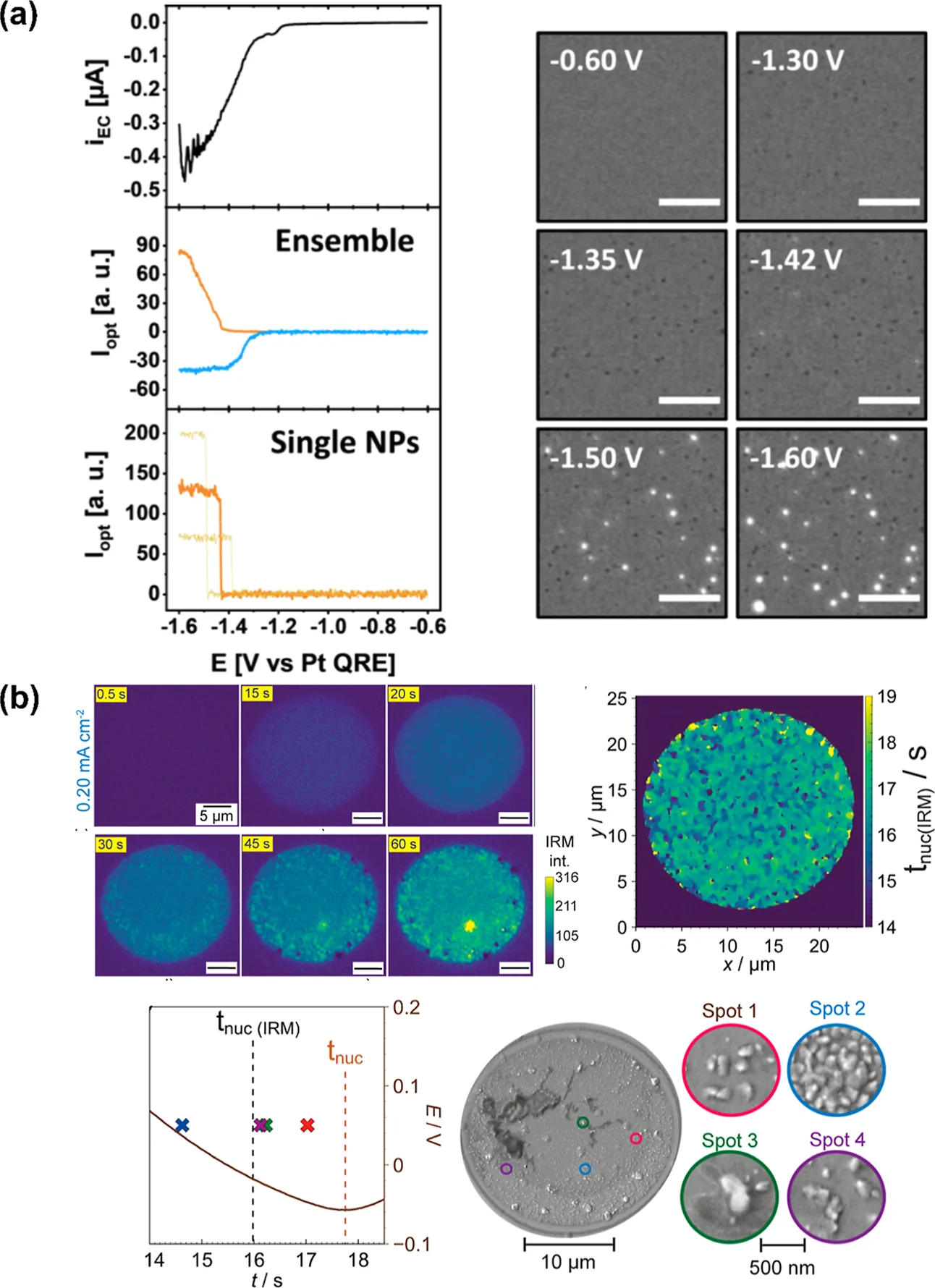
(a) Optical identification between competing reaction routes for Ni electrodeposition. Linear sweep voltammetry current–voltage response (top left) for Ni electrodeposition, within a scanning electrochemical cell microscopy (SECCM) spot on an indium titanium oxide semitransparent electrode, visualized by optical microscopy. Snapshots at different cell potentials are shown on the right and are used to extract ensemble (orange trace for bright-contrasted features, assigned to Ni deposits, appearing at more negative potentials; and blue trace for dark-contrasted features, assigned to Ni­(OH)_2_ deposits) and exemplary single-nanoparticle intensity profiles. Adapted with permission from Godeffroy, L. et al. Deciphering Competitive Routes for Nickel-Based Nanoparticle Electrodeposition by an Operando Optical Monitoring. *Angew. Chem. Int. Ed*. **2021**, 60 (31), 16980–16983 (ref 88). Copyright 2021 Wiley & Sons. (b) Optical monitoring of the nanoscale Li plating dynamics under battery system-relevant conditions. Snapshots (top left) from the optical recording of the Li electrodeposition area, on an Au semitransparent electrode, using galvanostatic SECCM, and the extracted map of nucleation time (top right). The analysis of the optical data returns an average nucleation time, across the microscale deposition area, earlier than the one determined from the galvanostatic potential–time trace (bottom left), with a spatial variation that is supported by SEM (bottom right). Adapted with permission under a Creative Commons (CC BY 4.0) License from Martín-Yerga, D. et al. High-Throughput Combinatorial Analysis of the Spatiotemporal Dynamics of Nanoscale Lithium Metal Plating. *ACS Nano* **2024**, 18 (34), 23032–23046 (ref 89). Copyright 2024 American Chemical Society.

Elsewhere, the coupled SECCM-IRM technique enabled the monitoring, by their electrochemical and optical signature, of individual Ag nanoparticles nucleating and evolving within the miniature cell at the end of the pipet probe.^90^ Different pipet aperture sizes resulted in different numbers of nanoparticles forming, down to single nanoparticles within the SECCM meniscus. The configuration, being sensitive to phase changes at the electrolyte (meniscus)–electrode interface, allowed the detection and spatiotemporal tracking of diffraction-limited entities. Analysis suggested that Ag nuclei diffuse and aggregate into nanoparticles, supporting nucleation–aggregation growth models.

The electrodeposition of Li, under battery system-relevant conditions, was investigated by combining SECCM with IRM inside a glovebox, under an Ar atmosphere (Figure 3b).^89^ The optical recording of the semitransparent Au film electrode led to nanoscale mapping of the Coulombic efficiency, highlighting areas of inactive Li accumulation during plating and stripping cycles. Additional galvanostatic experiments were performed at various current densities, reflecting the platform’s capacity for customization and parameter screening. These showed the optical intensity to be a more accurate (earlier) descriptor of the nucleation time, compared to the standard analysis of the galvanostatic potential–time curve, while also providing identification of spatial heterogeneities on the electrode surface and the resulting deposit morphologies.

SECCM has become a particularly powerful technique when combined with other analytical tools.^14,91,92^ The uptake of SECCM with integrated optics is expected to further increase as the use of colocated experimental procedures accelerates.^8^ Containment of the SECCM instrument inside a glovebox prompts additional synthetic capabilities and is useful for particular battery and energy storage possibilities which are at the forefront of some metal nucleation and kinetic studies, for example.^14^ Technique integration would also be beneficial to MCED-based methods, especially those that offer *operando* or in situ material characterization, but advances have typically focused on increasing design complexity or multiplexing current instrumentation.^8,37^

All in all, these studies highlight the significant progress that SECCM has made in single-particle metal nucleation at foreign substrates, which is not only of fundamental importance but also critical as this is the first step in the fabrication of 2D and 3D metal nanostructures that are considered next.

### 3.2 Two- and Three-Dimensional Metal Structure Fabrication

While SECCM has focused on achieving nanoscale resolutions to uncover fundamental mechanistic processes, MCED studies have typically implemented pipet aperture diameters between 500 nm and 500 μm and focused on multidimensional structure formation. We believe the mechanistic observations and technical advances from nanoscale metal nucleation via SECCM could usefully inform and guide microscale MCED structure fabrication. SECCM has occasionally been used for metal structure fabrication, as discussed throughout this section where appropriate, but the main focus is on MCED. Differences and similarities between related approaches are highlighted as appropriate.

Multidimensional fabrication via SECCM and MCED is typically realized in one of two ways: (i) continuous, by maintaining meniscus–surface contact while moving the pipet (e.g., retracting to produce vertical structures; Figure 4a); or (ii) layer-by-layer (or *voxel-by-voxel*—one *volume element* at a time), comprising of distinct sequential growth cycles (Figure 4b).

**Figure 4:**
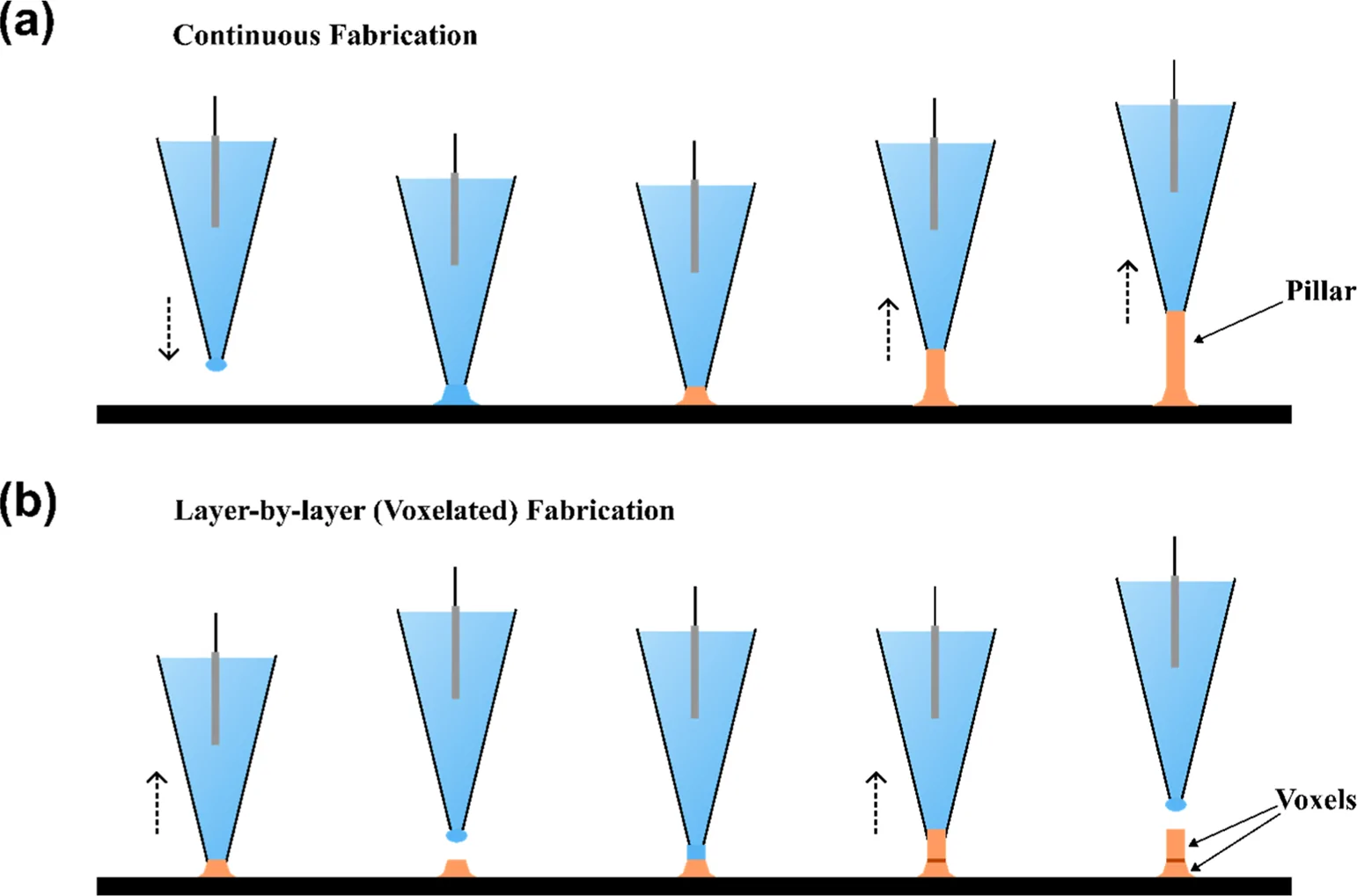
(a) Constant-contact material deposition (continuous fabrication) through scanning electrochemical cell microscopy (SECCM). A pipet approaches a surface to form a meniscus, and a potential is applied to trigger material deposition. The pipet is then retracted at a rate matching the pillar growth, maintaining a connection and leading to constant deposition. After the deposition is complete, the pipet can then be retracted faster or the potential switched, to end material deposition and release the pipet. (b) Layer-by-layer (voxelated) material deposition through SECCM. After a meniscus is formed, an initial step of material is deposited. The potential is then switched or the pipet retracted fast to end deposition and break the connection. The pipet then approaches the surface again, forming a meniscus on top of the previous deposition and depositing another layer. The pipet then once again retracts, and the cycle repeats until a deposit of desired height is formed. Note that all schematics are generated by authors and are not to scale.

Considering continuous fabrication, one of the earliest demonstrations of SPM-based MCED was reported by Iwata et al.,^93^ using shear force to detect meniscus contact when the shear-force oscillation amplitude decreased by 10% of its original value. Upon meniscus formation, a potential bias was applied between an Au wire inserted into the pipet and a cathodic Si surface to electrochemically reduce Cu­(II) to Cu(0) metal (Figure 5a). Chronoamperometry at a range of overpotentials in a hopping-mode protocol showed a dependence between deposition conditions, such as potential and deposition time, and the amount of Cu deposited. A constant-distance protocol was used to successfully fabricate two-dimensional lettering, “SU”, with a highly reproducible line width.

**Figure 5:**
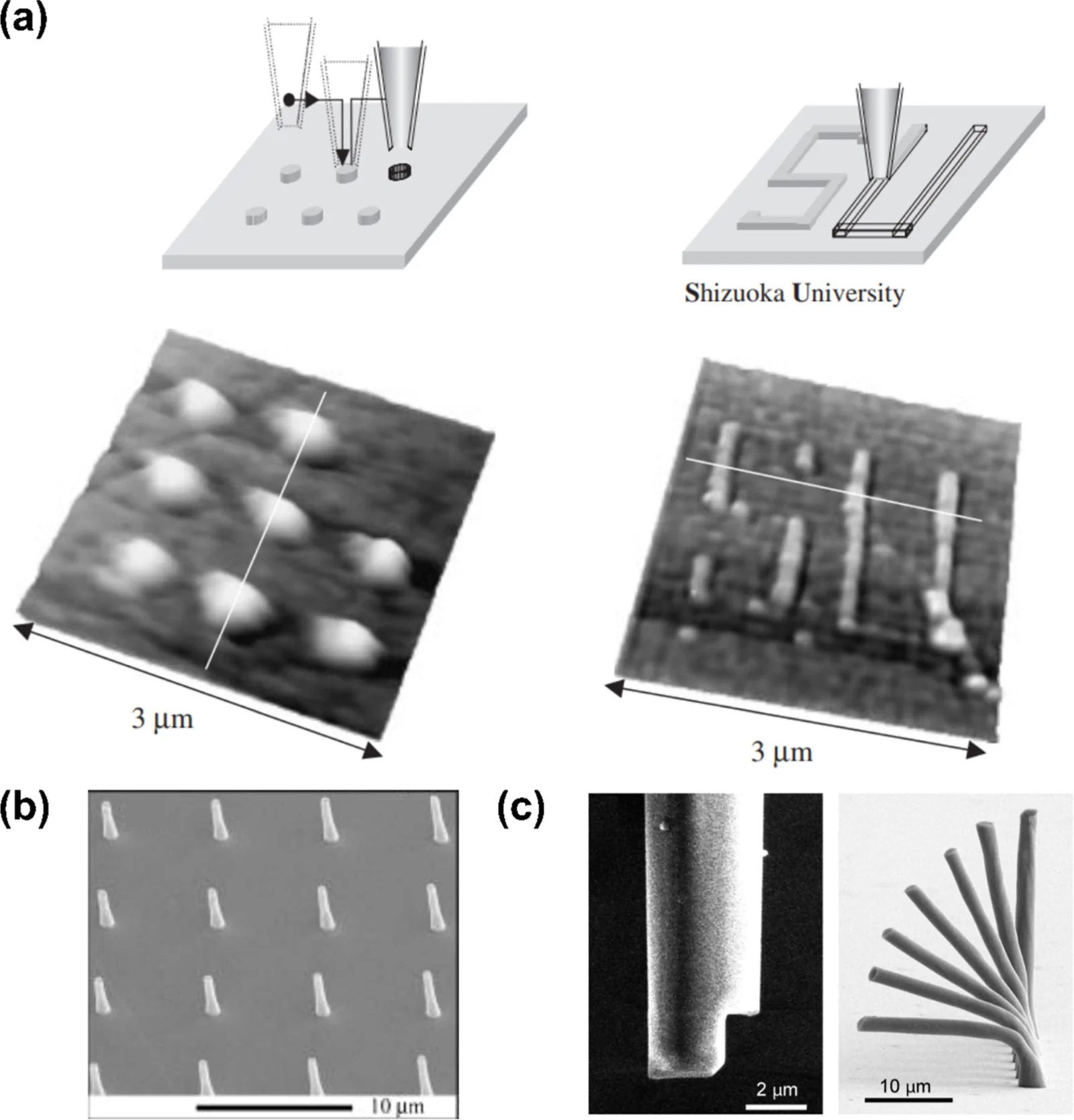
(a) Schematic representations (top) of the deposition procedure of Cu onto Si substrate and topographical images (bottom) of the resulting deposits. Both were obtained using a shear-force control technique, using a laterally oscillating pipet, with landing detection achieved by reduction in the oscillation amplitude when approaching a surface. The detection threshold for topographical imaging was reduction of the amplitude to 90% of its original value, and a reduction to 10% of the original amplitude was used for deposition. Each spot (bottom left) was deposited from a 0.01 M CuSO_4_ aqueous solution, using a potential of 1 V and a contact time of 1 s. The letters “SU” were deposited from the same solution, at a potential of 3 V and a deposition time of 60 s. Reprinted in part with permission from Iwata, F. et al. Nanometer-Scale Metal Plating Using a Scanning Shear-Force Microscope with an Electrolyte-Filled Micropipet Probe. *Japanese Journal of Applied Physics* **2004**, 43 (7S), 4482–4485 (ref 93). Copyright 2004 IOP Publishing. (b) SEM image of a Cu nanowire array formed on an Au/Si substrate from the SECCM-analogue, probe-based electrochemical deposition method. A 500 nm aperture probe containing 0.05 M Cu aqueous solution was used with a deposition potential of 0.4 V and a constant retraction speed of 250 nm s^–1^. Adapted in part with permission from Suryavanshi, A. P.; Yu, M.-F. Probe-based electrochemical fabrication of freestanding Cu nanowire array. *Applied Physics Letters* **2006**, 88 (8), 083103 (ref 94). Copyright 2006 American Institute of Physics. (c) (right) SEM images of Cu microwires fabricated at varying angles from a micropipet (left), using electrodeposition in the meniscus-confined electrodeposition method. Focused ion beam machining was used to cut a portion of the pipet nozzle, allowing for advanced patterning away from, then parallel to, the surface with a single probe. Reprinted in part with permission from Hu, J.; Yu, M.-F. Meniscus-Confined Three-Dimensional Electrodeposition for Direct Writing of Wire Bonds. *Science* **2010**, 329 (5989), 313–316. (ref 19). Copyright 2010 American Association for the Advancement of Science.

Subsequently, Suryavanshi and Yu^94^ introduced piezo-driven electronics into MCED-based instrumentation to monitor the pipet–surface distance with submicron resolution. In these studies, there was physical pipet–surface contact, increasing the risk of pipet and surface damage. The work demonstrated the fabrication of three-dimensional Cu nanowires on Cr/Au-coated Si surfaces. At a pipet retraction speed of 250 nms^–1^, Cu nanowires of 3 μm in length and less than 1 μm in diameter were fabricated from a pipet of a 500 nm opening diameter and visualized using SEM (Figure 5b). Nanopipets down to 100 nm in diameter were used to fabricate Cu nanowires approximately 200–250 nm in diameter and 10 μm in length. The study also highlighted the deposition of nanowires at tilted angles (up to 60°) to the substrate surface by synchronizing the motion of the pipet and surface during deposition.

In a similar experimental MCED-based approach, researchers attempted Pt nanowire deposition onto a Pt/Ir-coated substrate using nanopipets with aperture diameter down to 100 nm.^95^ Upon meniscus–surface contact, a potential bias of −1 V (vs Pt_QRCE_) was applied to the substrate, and the pipet was withdrawn at a speed of 50 nm s^–1^, producing a nanowire of ∼150 nm in diameter and 30 μm in length. In this study, the impact of relative humidity was explored, with lower relative humidities (<20%) creating frequent blockage events. The deposits were found to be of polycrystalline structure while two-point resistance measurements showed that their resistivity compared well to the bulk resistivity of Pt.

In a later work by the same group, a pipet-based MCED technique (coined “meniscus-confined 3D electrodeposition” by authors) was reported using micropipet apertures from several μm to 100 nm in diameter to develop Cu and Pt interconnects with dimensions down to ∼100 nm in diameter and >80 μm in length.^19^ The research showed that a ∼3 μm diameter pipet modified by FIB machining enabled stable meniscus formation at any angle between 0° (parallel to the surface) and 90° (normal to the surface, Figure 5c). The interconnects were electrically characterized showing similar resistance values to the expected bulk Cu wire. The study defined the growth rate of the nanowires as intrinsically limited by the diffusion-limited ionic current,^96^ proposing an increase in electrolyte concentration or use of a short-tapered micropipet to speed up deposition.

All the work considered thus far has demonstrated the use of constant-contact mode for three-dimensional fabrication where meniscus–surface contact is maintained as the pipet is retracted at a rate equal to local growth.^38^ A disadvantage to this strategy is that pipet clogging and meniscus detachment are likely when the retraction speed is not well-matched to the growth rate.^97^ Layer-by-layer fabrication was developed to address this by growing material volume elements^97^ (e.g., in voltammetric cycle–retraction pairs) to form stacked architectures reminiscent of commercial extrusion-based 3D printing techniques.^98^ These voxelated methods do have limitations, in particular: (i) producing structures slower than constant-contact methods and (ii) ones that typically show ribbed surface textures.^97^

Voxelated deposition of Cu nanowires has been achieved via an MCED technique referred to as 3D metal microprinting.^99^ Glass pipets down to ∼250 nm in diameter were used to electrodeposit CuSO_4_ (500 mM) onto an Au surface, resulting in high aspect ratio metal wires of ∼200 nm in diameter with an average voxel height of 100 nm. This study also introduced a parallel printing process using a 1 × 10 pipet (20 μm in diameter) array, enabling the fabrication of ten identical microstructures at once (Figure 6a). While this work only reported the parallel printing process with pipet apertures of 20 μm, which is far beyond the scope of SECCM, the initial fabrication of nanowires clearly demonstrates the shared framework between SECCM and pipet-based MCED techniques. This highlights the simplicity of changing pipet for one with a smaller aperture opening on the same experimental instrumentation to access different scale dimensions.

**Figure 6:**
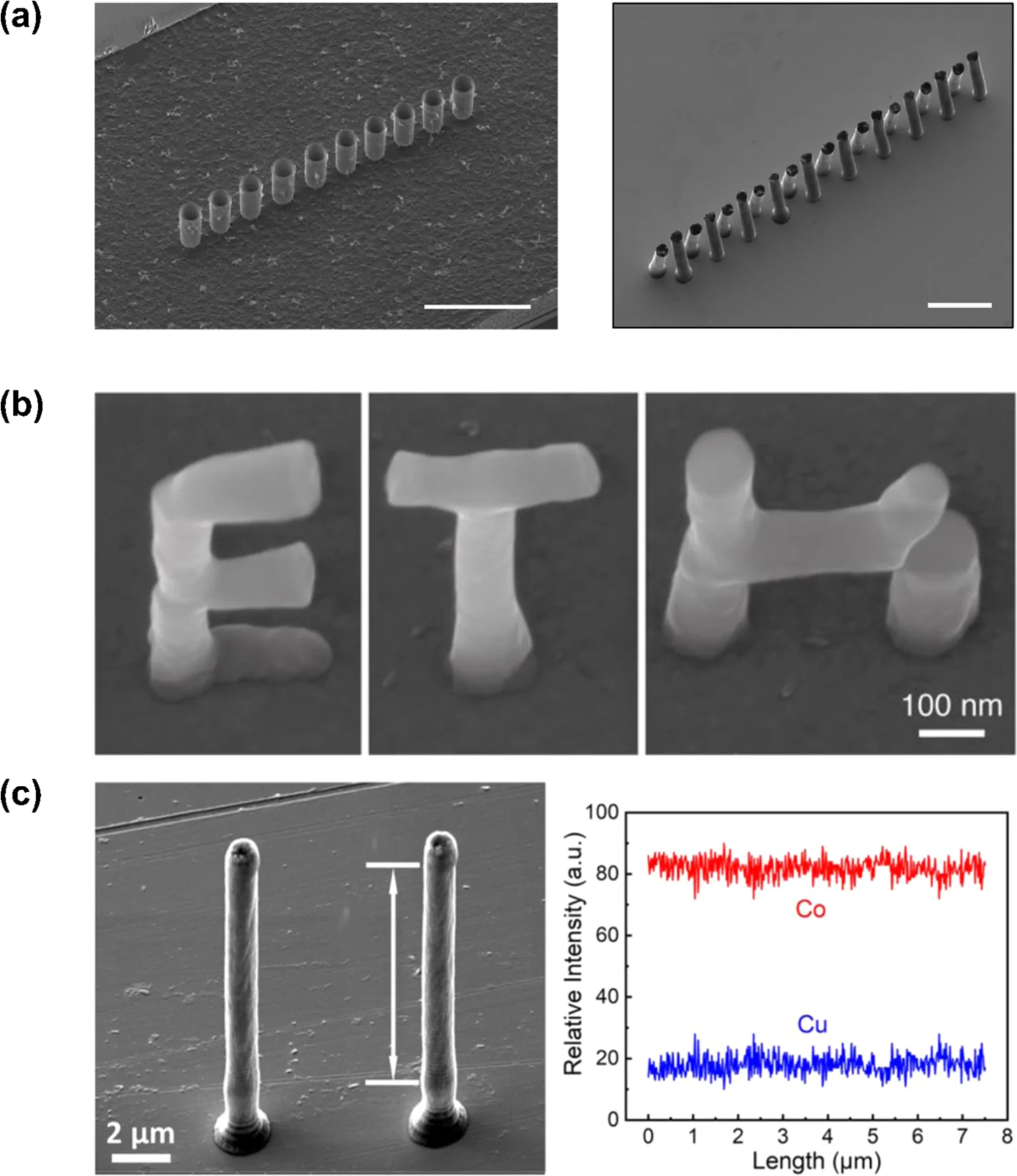
(a) SEM images showing 1 × 10 microfabricated nozzle array (left, scale bar represents 150 μm), used to deposit the array of tilted Cu wires in parallel on an Au-coated substrate (right, scale bar represents 100 μm). Reprinted in part with permission from Lin, Y.-P.; et al. Parallel Process 3D Metal Microprinting. *Advanced Materials Technologies* **2019**, 4 (1), 1800393. (ref 99). Copyright 2018 Wiley & Sons. (b) Cu structures showing nanoscale letters “ETH”, deposited on an Au substrate. Reprinted in part with permission under a Creative Commons (CC BY-NC-ND 4.0) License from Hengsteler, J.; et al. Bringing Electrochemical Three-Dimensional Printing to the Nanoscale. *Nano Letters* **2021**, 21 (21), 9093–9101 (ref 37). Copyright 2021 American Chemical Society. (c) SEM image (left) showing Co/Cu alloy wires deposited on an Au substrate and the concentration profiles of the two metals (right) obtained by energy-dispersive X-ray spectroscopy. Reprinted in part with permission under a Creative Commons (CC BY-NC-ND 4.0) License from Lei, Y.; et al. The Composition and Magnetic Property of Co/Cu Alloy Microwires Prepared Using Meniscus-Confined Electrodeposition: Effect of [Co2+], [Cu2+] Concentration at the Tip of the Meniscus. *Journal of The Electrochemical Society* **2021**, 168 (11), 112507 (ref 102). Copyright 2021 IOP Publishing.

In a recent advancement to voxelated Cu electrodeposition, researchers employed an MCED-based approach analogous to SECCM which authors cited as meniscus-confined electrochemical additive manufacture.^37^ This technique enabled tunable voxel size on-the-fly by controlling the potential between two set points with a 45 nm pipet. Overall feature size was shown to increase linearly with cathodic potential from 59 nm (±3 nm) to 106 nm (±3 nm). Individual voxel height was shown to increase from 3 nm (±0.1 nm) to 10.7 (±1.1 nm). It was also noted that the printing rate was shown to exponentially increase from 6.6 nm s^–1^ to 69.7 nm s^–1^. In the same work, an impressive example of freestanding letters “ETH” was illustrated using a 45 nm diameter pipet to produce consistent vertical and horizontal feature diameters of 90 nm ± 11 nm (Figure 6b). Without the need for specialized pipet fabrication, this voxelated method enabled printing tilted structures at any angle. Despite these innovations, the authors described that printing angles >45° remained challenging, noting incomplete bridging in the letter “H” due to misalignment. To investigate resolution limits using electrochemical meniscus-confined approaches, reproducible pillar structures were fabricated with 1.6 and 2 nm aperture pipets. It was discovered that features sizes below 25 nm were difficult to deposit even with ultrasmall probe sizes, emphasizing an increase in pipet blocking and a requirement for faster electronic feedback to enable fabrication at these scales.

Direct use of single-barrel SECCM for Cu electrodeposition has been reported across tilted substrates.^100^ The surface tilt was elucidated through initial landing points in a prescan, reminiscent of bed-leveling programs commonly used in commercial 3D printing techniques.^101^ In this work, Cu depositions using both continuous contact and voxelated printing methods were fabricated to validate the surface tilt elucidation. While dual-barrel systems offer more flexibility to pattern on rough or uneven topographies (see above), these single-barrel protocols provide practical solutions on well-controlled surfaces without the need for complex dual-barrel electronics.

In a distinctive application of constant-contact meniscus-guided fabrication, Co/Cu alloy microwires were fabricated from a single electrolyte solution in an MCED approach synonymous with single-barrel SECCM.^8^ A cobalt-rich solution was chosen to counteract the increased deposition rate of Cu compared to Co under identical potential conditions. An electrolyte-filled micropipet (1.4 μm diameter) was held at varying potentials versus a Ni/Au-coated Si substrate and slowly retracted in a constant-contact manner to produce three-dimensional alloy microwires. Characterization of the resulting alloy microwires was achieved by EDS, selected-area electron diffraction, TEM, and XPS, confirming the fabrication of homogeneous and polycrystalline alloy microwires (Figure 6c).

The influence of relative humidity on microwire morphology was also explored in this research.^8^ Despite improvements in growth rate, lower relative humidities were shown to have an adverse effect on deposition uniformity. These studies allowed the optimal relative humidity for the fabrication of uniform microwires to be established, but relative humidity was also found to affect the elemental ratio of Cu to Co in the depositions—with higher increased relative humidities showing a decrease in Cu content. This work highlights the environmental control required to fine-tune deposit composition in multimaterial depositions and emphasizes the need for environmental conditions to be further explained more generally.

Voxelated approaches are gaining increasing attention in three-dimensional metal deposition studies compared to constant-contact modes. With the development of faster piezoelectric systems, the advantages of layer-by-layer deposition are outweighing its limitations. For example, the introduction of periodic deposition cycles into a dual barrel pipet-based MCED technique (coined “intermittent MCED” or “iMCED” by authors) restricted meniscus lifetime to milliseconds: (i) preventing pipet clogging, (ii) acting as positional feedback; and (iii) increasing deposition speed to values comparable to continuous growth methods.^103^

While these voxelated methods prove to be superior to continuous growth modes in the *z*-axis, constant-contact methods still serve a necessary purpose in two-dimensional deposition. Two-dimensional metal growth has had limited exploration using SECCM- and MCED-based techniques. Other techniques such as nanolithography are already well-established for these purposes at ultrahigh resolutions and feature intricacy.^104^ Therefore, the unique capabilities of SECCM- and MCED-based techniques for metal deposition, including nucleation growth and access to the third dimension, have been the focus of studies on metal systems. However, this is not true for all systems, as will be explored in the following sections.

## 4 Confined Molecular Material Deposition

We now turn to molecular material deposition and the chemical modifications of surfaces as a prelude to polymer synthesis which is discussed subsequently. SECCM has proved to be a key technique for modifying surfaces locally by various means, including adsorption, surface–chemical bond formations, and reactive grafting.

In early work, dual-channel SECCM was used to functionalize graphite surfaces with absorbed anthraquinone-2,6-disulfonate (AQDS).^39^ Chemically functionalized carbon surfaces have a myriad of applications and their study is important for identifying key surface sites. Here, researchers chose HOPG with different densities of step edges (spanning more than 2 orders of magnitude difference) to show that the basal surface dominated the electrochemistry of AQDS. By implementing a fast scan cyclic voltammetry process, it was also possible to follow the adsorption process in real time. Surface functionalization can also be promoted by a chemical reaction leading to chemical bonding to the surface.^105^ The use of SECCM to promote and investigate such processes is exemplified by studies of diazonium modification of carbon surfaces,^106^ where arrays of diazonium-functionalized spots on HOPG were created by applying different driving forces and durations, with the resulting films characterized by AFM and Raman spectroscopy.

Self-assembled monolayers (SAMs) are a popular method to modify surface functionality. The formation of a SAM has been directly monitored using SECCM through the investigation of alkylthiol–gold interactions.^10,107^ SAM formation progressively diminished the intensity of the electrochemical signal while repeated measurements helped map the rate and density of the SAM layer. The same methodology permitted the controlled reductive desorption of the SAM layer. By cyclic voltammetry profile analysis, shorter alkanethiol chains were found to desorb readily compared to longer chains, owing to the decreased effect of van der Waals interactions.^107^ Irregular profiles were observed when less dense SAMs formed along Au surface grain boundaries.

## 5 Polymer Synthesis and Deposition

### 5.1 One-Dimensional Polymer Synthesis and Deposition

Electrochemical polymerization proceeds via the generation of reactive intermediates by oxidative or reductive pathways. Compared to thermal or chemical methods, electrochemical initiation can offer superior control over polymer chain length, morphology, and architecture, due to its capacity for dynamic and responsive control over the electric field.^108^

Oxidative polymerization typically proceeds via formation of radical cation intermediates by directly exploiting the redox behavior of suitably active monomers. The synthesis of conjugated (or *conducting*) polymer films, such as polyaniline,^109^ polypyrrole,^110^ and poly­(3,4-ethylenedioxythiophene) (PEDOT),^111^ has been extensively reported using oxidative polymerization techniques. Precise spatiotemporal control of film formation and the ability to tune the conductivity of polymer films are two advantages of this electrochemically mediated polymer synthesis. These polymers have shown promising potential in the applications of flexible bio­(electronics),^112,113^ (bio)­sensing,^114,115^ and energy-storage applications.^116–118^ Though less common,^108^ the polymerization of vinyl monomers to produce insulating polymer films is accessible through indirect electrochemical pathways.^119,120^ For example, by generating radicals in the presence of vinyl monomer species, free-radical polymerization (FRP) can be initiated.^121^

Under electrochemical control, each stage of polymerization is sensitive to applied potential,^122,123^ deposition time,^124^ and local chemical environment,^125^ including substrate properties,^126^ electrolyte composition,^127^ and counterion influence.^128^ The complex interplay of these variables can lead to many different deposition outcomes, affecting the quality and reproducibility of synthesized polymers, including film morphology,^129^ thickness,^130^ and electrical conductivity.^131^ SECCM and MCED techniques offer a means to explore the effects of electrochemical parameters on polymer synthesis with minimal reagent use and limited waste compared to macroscale methods.

Electropolymerization of a polypyrrole array by SECCM has been used to explore the viability and meniscus stability of SECCM-based electrodeposition in aprotic media.^132^ Using a micropipet of 1.7 μm in diameter filled with a pyrrole in propylene carbonate solution, identical potential–time waveforms were applied to each landing site on an Au substrate, generating highly reproducible current–time curves, and corresponding polypyrrole depositions, with an average charge of 6.5 ± 0.8 pC. Characterization by SEM revealed that the polypyrrole depositions were visible as uniform black “dots” surrounded by a circular halo around ∼2.5 μm in diameter, representing the entire wetted footprint of the pipet.

More recently, the impact of anionic dopant incorporation into polypyrrole has been investigated using electrochemical additive manufacturing based on meniscus-guided deposition.^133^ Although the in-house-developed MCED technique used in this study is not directly equivalent to SECCM, similarities in experimental design and analysis provide useful points of comparison and context capable of guiding SECCM deposition protocols. In the work, a pressure-controlled polyethylene micropipet (∼200 μm in diameter) filled with a solution of pyrrole and anionic dopant was used to electropolymerize pyrrole onto a stainless-steel substrate under galvanostatic conditions (current density of 1 mA cm^–2^). Factors such as monomer concentration, applied voltage, and dopant structure were varied to correlate experimental parameters to deposition outcomes. Deposition morphology was found to vary greatly between dopant structures and deposition techniques with the work highlighting drastic differences between polypyrrole films deposited with meniscus-guided and macroscale approaches.

Counterion influence and supporting electrolyte composition can uniquely interact with polymer synthesis mechanisms to affect the resulting polymer composition and morphology.^128^ Lasting salt residues remaining on the substrate surface after pipet landing has been reported in SECCM.^52,134^ To improve consistency and reproducibility between consecutive depositions of polypyrrole, researchers have introduced a cleaning step to the typical SECCM hopping protocol.^135^ By alternating the deposition potential between the polymerization potential of pyrrole and a potential where no reactions occur (i.e., maintaining the approach potential), tip blockage and side product formation could be minimized while improving meniscus stability. The work confirmed these findings for different electrolyte compositions and chemical environments, noting electrolyte-specific embedding effects within the polymer matrix, which were effectively removed with a distilled water rinse. Importantly, this work demonstrated the polymerization of pyrrole onto rough, maze-like, boron-doped carbon nanowall substrates using voxelated deposition (four cyclic voltammetry cycles), overcoming typical challenges that uneven surfaces pose.

In an example of the indirect electrochemical polymerization of vinyl monomers, researchers have demonstrated the use of SECCM to drive the Cu-mediated reversible deactivation radical polymerization (RDRP) of acrylamide derivatives (Figure 7).^119,136^ In both cases, this was achieved through surface modification by SAM formation (as discussed in the previous section) including the initiator species onto an Au substrate. In this experimental design, polymers with brush-type architectures anchored to the monolayer surface are formed. Localized surface modification with polymer brushes has promising impact on a range of applications in biotechnology such as nonspecific fouling and drug encapsulation.^137^

**Figure 7:**
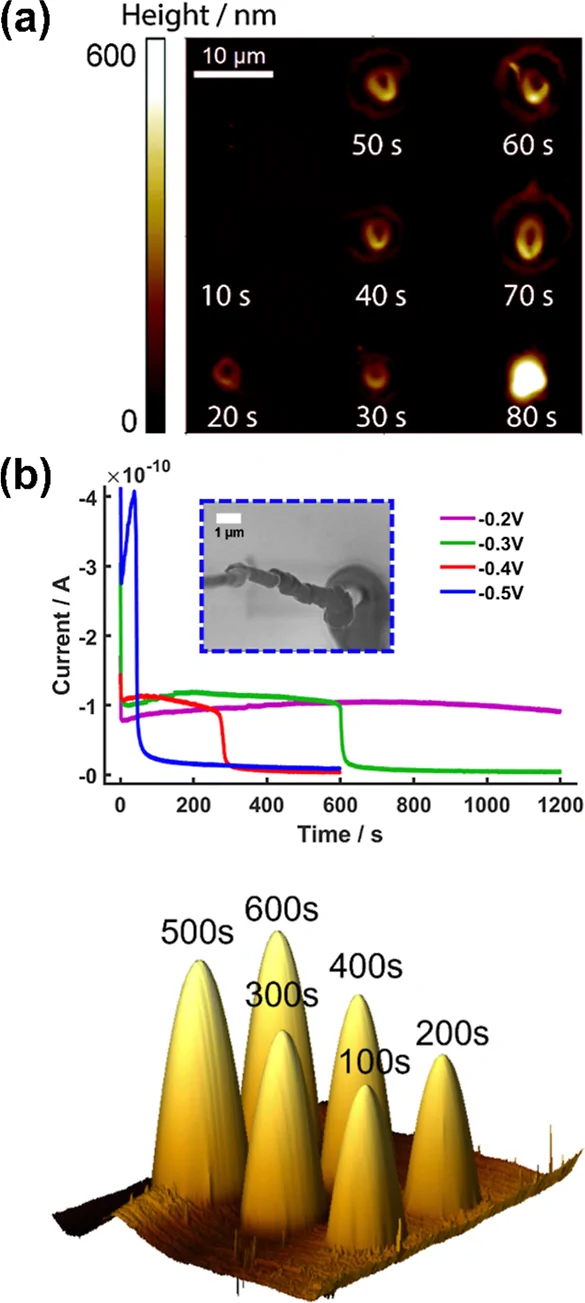
(a) Topographic AFM image of poly­(*N*-hydroxyethyl acrylamide) deposits formed on an OH-terminated self-assembled monolayer (SAM), supported on an Au electrode. The deposits were formed by electrochemically activating a Cu­(II)­Cl_2_/tris­[2-(dimethylamino) ethyl] amine (Cu­(II)­Cl_2_/Me_6_TREN) catalyst in the tip, forming Cu­(I)­Cl/Me_6_TREN, which then polymerizes the monomer (*N*-hydroxyethyl acrylamide), also present in the solution. Deposits of increased height were formed by increasing the contact time of the tip on the surface, from 10 to 80 s. Reprinted in part with permission from Oseland, E. E., et al. Surface patterning of polyacrylamide gel using scanning electrochemical cell microscopy (SECCM). *Chemical Communications* **2016**, 52 (64), 9929–9932 (ref 136). Copyright 2016 Royal Society of Chemistry. (b) (top) Current–time traces of acrylamide polymerization, achieved with electrochemically mediated atom-transfer radical polymerization in scanning electrochemical cell microscopy on a SAM-coated Au substrate. Each trace corresponds to a different surface potential during the polymerization. At more negative potentials, blocking of the tip occurs, causing sharp current drop-offs. A SEM image is included of a poly­(acrylamide) feature formed from an applied potential of −0.5 V. (Bottom) Three-dimensional representation of an AFM image showing the heights of poly­(acrylamide) deposits formed with an applied potential of −0.15 V at different reaction times (100–600 s). Adapted with permission under a Creative Commons (CC BY 3.0) License from Mohammed, M., et al. Localized polymerization of acrylamide using single-barrel scanning electrochemical cell microscopy. *Chemical Communications* **2023**, 59 (73), 10992–10995. (ref 119). Copyright 2023 Royal Society of Chemistry.

Together, the works discussed demonstrate the successful synthesis of polymeric materials via SECCM and related meniscus-guided techniques across multiple reaction pathways in different chemical environments. Divergences from the macroscale electropolymerization behavior highlight that confined electropolymerization cannot be considered with the same assumptions as macroscale processes.

Building on these principles, patterned deposition of polymers is achievable using meniscus-confined techniques, as discussed in the next section. Compared to other polymer patterning methods, its nanoscale resolution capabilities distinguish SECCM from inkjet and extrusion-based techniques, while electrochemical initiation removes the need for optically clear inks required in techniques such as nanolithography and two-photon polymerization.^108,138,139^

### 5.2 Two- and Three-Dimensional Polymer Structure Fabrication

Constant-contact deposition modes hold important relevance in the multidimensional deposition of polymer systems. With the use of dual-barrel pipets, the pipet–surface distance can be automatically adjusted to compensate for surface roughness to access two-dimensional polymer patterning across insulating surfaces.^38^ SECCM is compatible with a diverse selection of polymers and precursor materials, including many of those considered unsuitable for other well-established patterning techniques.^17^ Three-dimensional polymeric structures can proceed via SECCM by either of two mechanisms presented in metal deposition (i.e., voxelated or continuous).

The electropolymerization of pyrrole via meniscus-guided polymerization—a technique functionally equivalent to single-barrel SECCM^35^—was reported to facilitate the successful fabrication of freestanding nanowires (≥50 nm) using a constant-contact retraction method.^140^ Despite the use of three-axis stepping motors for positioning (unlike the superior piezoelectric actuators usually described in SECCM instrumentation), researchers achieved a positioning accuracy of 250 nm, demonstrating this through the fabrication of various structural architectures, including lettered imagery, nanocandles, nanobranches, and nanoarches (Figure 8a). Additionally, the work highlighted the positive correlation between key deposition parameters, such as retraction time and electrolyte flow rate, and nanowire dimensions, like height and width.

**Figure 8:**
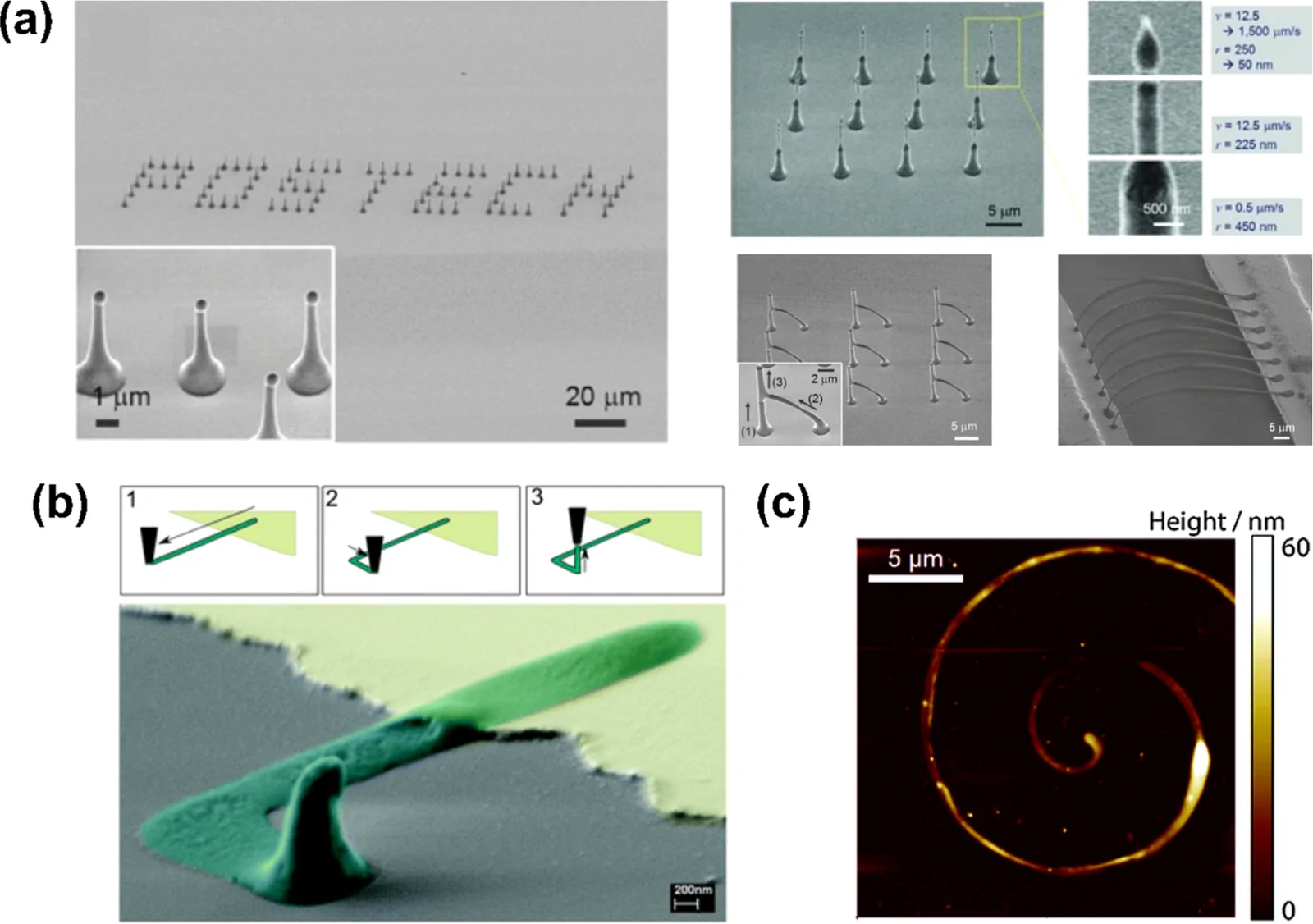
(a) SEM images of polymer nanowires forming the letters “POSTECH” (left), nanocandles (top-right), and nanobranches (bottom-middle) on Pt surfaces, and nanoarch bridges (bottom-right) on Cu grids. Adapted with permission from Kim, J. T. et al. Three-Dimensional Writing of Conducting Polymer Nanowire Arrays by Meniscus-Guided Polymerization. *Advanced Materials* **2011**, 23 (17), 1968–1970. (ref 140). Copyright 2011 Wiley & Sons. (b) SEM image with false color added of conductive polyaniline (green) fabricated from a conductive (Au) onto insulating (gray) substrate, along with a schematic diagram of the deposition. Reprinted with permission from McKelvey, K. et al. Meniscus confined fabrication of multidimensional conducting polymer nanostructures with scanning electrochemical cell microscopy (SECCM). *Chemical Communications* **2013**, 49 (29), 2986–2988 (ref 38). Copyright 2013 Royal Society of Chemistry. (c) Topographical AFM image of poly­(*N*-hydroxyethyl acrylamide) deposits formed with scanning electrochemical cell microscopy on a self-assembled monolayer-coated Au substrate. Reprinted in part with permission from Oseland, E. E., et al. Surface patterning of polyacrylamide gel using scanning electrochemical cell microscopy (SECCM). *Chemical Communications* **2016**, 52 (64), 9929–9932 (ref 136). Copyright 2016 Royal Society of Chemistry.

In 2013, McKelvey et al.^38^ published the first example of electropolymerization via SECCM. Two-dimensional line patterns of polyaniline (with a width of ca. 1 μm) were fabricated under potentiometric and galvanostatic conditions using dual-barrel SECCM. In each case, the research demonstrated that varying potential or current controlled the rate and density of polyaniline deposition. This work also shared a rare example of electropolymerization by SECCM across an insulating substrate. Dual-barrel positional feedback enabled deposition to begin on a conductive Au surface, and then, move laterally across onto a nonconducting glass surface (Figure 8b). Retraction of the pipet from the nonconducting surface at a rate equal to deposition growth rate resulted in a three-dimensional polyaniline tower. The electrical contact maintained between the pipet and Au working electrode surface confirmed the polymerization of conductive polyaniline. This work remains an exemplar study in the capabilities of dual-barrel configurations for polymer fabrication.

The electrocatalytic RDRP approach discussed earlier in this section has successfully been used for polymer patterning with dual-barrel SECCM.^136^ The spiral-shaped fabrication of insulating poly­(2-hydroxyethylacrylamide) brushes using a 200 nm pipet diameter was achieved on a SAM-modified Au surface (Figure 8c). The depositions were characterized using XPS which showed results consistent with polymer deposition. In this approach, three-dimensional growth into the *z*-axis is limited, as chain propagation depends on the diffusion of the catalyst to and from the surface. As denser and longer brushes form, the activation of the catalyst becomes increasingly difficult. However, with less-dense brush formation, the deposition potential and time can control vertical brush growth into the *z*-axis.

From direct^38,140^ and indirect^119^ reaction pathways to monomer compatibility and surface grafting modifications,^136^ the versatility illustrated in the works above exposes the breadth of deposition reaction conditions accessible using SECCM and MCED techniques for polymer synthesis and deposition. This adaptability, inherent to their shared experimental framework, defines the distinct capabilities of electrochemical scanning probe techniques in their use for submicrometer polymer patterning from extrusion-based and nanolithography techniques.

Despite these possibilities, MCED research has had less focus on polymeric systems compared with SECCM. Polymer characterization at nanoscale electrode surface regions, and thus, at subnanoliter volumes of reaction product,^141^ has explained resistance in uptake. The increase in operando combinatorial techniques into SECCM and MCED,^8,14,91,92^ which is expected to improve characterization limitations in all studied systems, has already been discussed in depth. With a class of materials such as polymers, recognized for their application-specific structure–function capabilities,^8,142^ we hope that broader adoption of tailored nanoscale polymer deposition is realized in the near future, especially within MCED, material, and polymer chemistry communities.

Advances in nanoscale polymer patterning are expected to contribute to a range of potential applications, including in energy storage applications for solid electrolytes with ultrahigh surface areas, micro- and nanoelectronic circuit applications, and as removable supports for submicrometer additive manufacture to access *almost* limitless structure geometry.^143,144^

Still, polymer synthesis and growth can cause severe distortion to meniscus geometry and stability.^145^ Tip blockage is severe in polymer synthesis using pipet-based techniques.^135^ Growth into the pipet is common, and precautions often rely on limiting meniscus contact time, silanization of pipet tips before use, and precipitation of solid polymer from the liquid meniscus electrolyte at the three-phase boundary (i.e., the *meniscus–air–surface* interface).^52,53,146^

## 6 Evaporative Deposition

The experimental approach in all of the works discussed so far used electrochemistry to drive reactions, with subsequent deposition of the reaction product. However, SECCM and MCED platforms can be used to drive evaporative deposition of components within the electrolyte solution. Most examples in the literature use a colloidal ink dispersion to deposit functional materials, including metallic nanoparticles,^147^ polymer composites,^148,149^ and functional nanomaterials.^150^ While not the focus of this Perspective, its reference was deemed important for completeness since future work in this area may involve the combination of electrochemical with evaporative (environmental) control.

Au nanoparticles have been delivered to surfaces from colloidal inks with evaporative deposition by a microscale meniscus-guided technique belonging to the MCED family.^147^ Au nanotips, with a width in the order of ∼100 nm, were fabricated onto a Si substrate from 5–10 μm diameter glass pipets. Expected applications of the nanotips, as stated by the authors, included a variety of nano-optical and analytical applications (e.g., tip-enhanced Raman spectroscopy (TERS), scattering-type scanning near-field optical microscopy (s-SNOM), scanning tunneling microscopy (STM)), and other forms of nanoscale optical, materials, and surface characterization.

Polymer composites, such as poly­(methyl methacrylate)/polypyrrole and poly­(3,4-eythlenedioxythiophene)/poly­(styrenesulfonate)—more commonly known as PMMA/PPy and PEDOT/PSS, respectively—have been of high focus to evaporative deposition by meniscus-guided techniques. Hollow PMMA/PPy microtubes have been fabricated by an evaporative MCED approach with no electrochemical data acquisition or control. The manufacture and device integration of these microtubes into individually addressable gas sensors has been published for more than a decade.^148^

One year before the first publication of SECCM-based deposition,^38^ nanoscale three-dimensional writing of PEDOT/PSS on a Pt substrate was achieved by evaporation of a polymer meniscus hanging from a nanopipet.^151^ Stretchable nanoarches (∼400 nm in diameter) demonstrated successful device integration in photoswitches and electrochemical transistors.

The first example of PEDOT/PSS by SECCM-based evaporative deposition was shared by Zhang et al.^152^ who described the use of a homemade variant of SICM to produce flexible pillars between 50 and 5000 μm. In the same study, a dual-barrel configuration of the instrumentation was used to measure the electrochemical activity of the pillars post-deposition. Notably, recent work by the same group^153^ has shared an interesting biological application for the fabrication of PEDOT/PSS nanowires by describing their integration into a heart-on-a-chip platform, serving as displacement sensors for real-time cardiac tissue contractile force measurements.

These works show the extension of SECCM and MCED platforms beyond electrochemical deposition, exemplifying the possible versatility of materials deposited by using these techniques. Functional integration into analytical devices and sensors shows the practical functions and industrial applicability of meniscus-guided manufacturing techniques.

## 7 Challenges and Opportunities

Despite its versatility, there are some technical aspects that need implementation to promote the broader adoption of SECCM and related meniscus-confined techniques for nanofabrication. In some situations, tip fouling, meniscus instability, and electrowetting can complicate reproducible nanoscale fabrication.^52^ This section revisits and expands upon some of the issues discussed in this Perspective to guide future experimental designs.

Certain assumptions are typically accepted during SECCM and MCED experimental design and data analysis, including (i) a stable and consistent meniscus geometry, (ii) a controlled atmosphere, and (iii) evaporation being negligible and having little effect. However, for deposition and fabrication, meniscus dynamics may critically influence deposition uniformity and growth rates.^154^ Environment, evaporative effects, pipet geometry and distance from the surface, electrochemical parameters, and mass transport, interact to determine the deposit shape, height, and morphology. Highly stable menisci produce uniform, controlled, and repeatable deposits.^155^

Pipet silanization and the addition of surfactants have been used purposely to influence meniscus geometry, mitigate uncontrolled wetting, and reduce pipet blocking, by chemically modifying the hydrophobicity of the pipet or surface.^134,156^ However, it is important to emphasize that material fabrication, deposition, and dissolution intrinsically disrupt and transform meniscus geometry during the surface modification process.^145^ Experimental investigations to control atmospheric conditions and optimize meniscus consistency have provided valuable knowledge to guide scanning probe deposition experiments,^157–159^ but further work in this direction is needed.

In practice, meniscus wetting may not always be consistent even under apparently ideal conditions.^145^ In fact, the meniscus is dynamic and may change geometry depending on the surface energy, humidity, electrolyte composition, and contact time.^145^ For fabrication, this affects the deposit dimensions, morphology, and spatial resolution. Analytical models and finite element simulations can be useful for describing deposition behavior,^160,161^ furthering our understanding of surface wetting, the impact of growth from within a meniscus, and the underlying mass transport mechanisms that define some rate-limiting deposition kinetics.

Another factor that may impact reproducibility and scalability is that commercial thermal and laser pullers for pipet preparation need to be kept maintained and well-tuned to prepare pipets with consistent geometry. Even so, to elucidate mechanistic and kinetic information from SECCM studies—as has been the aim of numerous metal nucleation studies that have been highlighted—exact pipet and meniscus geometry must be known.^45,162^ Therefore, high-resolution microscopy characterization of pipets is advised before (and after, where possible) deposition experiments.^45^

The introduction of IRM-integrated SECCM enables operando monitoring of deposition dynamics and the three-phase boundary, and correlation with time-dependent electrochemical signatures from experiments. This is particularly pertinent as retraction dynamics have been evidenced to significantly increase the deposit diameter.^45^

Contamination is another consideration in meniscus-confined deposition experiments. Leached Ag^+^ ions from the QRCE material, glass etchants (e.g., for high-pH aqueous solutions), and residual organics from electrolyte preparation, are some potential contamination sources.^163,164^ Ag/AgCl QRCEs based on coated silver wires are widely used in SECCM and their stability in a range of aqueous solutions over prolonged times (>6.5 h) has been investigated in detail to define the time window of operation alongside other considerations such as placement within the pipet.^165^ Care is particularly needed in reactive systems,^89^ where contamination could affect the primary outcomes of interest, including nucleation thresholds and deposit composition. Among other mitigations, a leakless Ag/AgCl can be used in place of a typical QRCE, if needed.^166^

A key feature in the application of SECCM for fabrication lies in its integration with complementary characterization techniques to analyze the structure and chemical composition of deposited materials.^167–169^ Workflows that enable automated feature matching and alignment between electrochemical and characterization data sets could accelerate the extraction of structure–function relationships and facilitate high-throughput studies—an aspect inherently supported in SECCM implementations.

In situ integration of spectroscopic methods can offer insight into compositional evolution, reaction intermediates, and degradation pathways.^170–173^ Coupling SECCM with Raman spectroscopy,^168^ X-ray diffraction,^169^ or mass spectroscopy,^92^ enables molecular and crystallographic analysis, even on the small scales typically associated with SECCM. Specialist techniques such as tip-enhanced Raman spectroscopy,^174^ nanoscale-Fourier transform infrared spectroscopy (nano-FTIR),^175^ or secondary ion mass spectrometry,^176^ may feature in the effort toward elemental characterization of organic deposition features. The development of a standardized, comprehensive workflow comprising SECCM and characterization techniques is essential to unlocking its full potential for nanoscale soft material fabrication.

Further coupling of SECCM with machine learning-based algorithms and additional characterization techniques such as mass spectrometry could facilitate real-time spectrum interpretation and automated product identification, enabling the screening of reaction products and intermediates in complex materials systems. While such automation has been successfully demonstrated for other SPM techniques, such as SICM, AFM, or STM,^177–179^ early steps toward “smart” SECCM routines are now being made. Methodologies have been demonstrated to estimate the number of nanoparticles deposited in a single spot from the overall electrochemical current and the current measured for an individual nanoparticle.^180^ A targeted SECCM approach has been realized using optical imaging of the area of interest and mapping of coordinates, achieving sequential landings on nanoparticle clusters without scanning an entire area of the supporting electrode, improving efficiency and resources utilization.^181^ Automation is also possible through long-range modifications with programmable scan routines or robotic mapping systems.^182^

In this Perspective, we have sought to provide experimental recommendations to guide future meniscus-confined electrochemical deposition. Among the issues considered, we have noted that operando and in situ characterization techniques can quantify information about deposition feature properties and meniscus dynamics. Dual-barrel configurations excel for deposition onto surfaces (irrespective of substrate conductivity) and enable multidimensional structure fabrication with active surface–meniscus distance feedback. Constant-contact deposition modes complement hopping-mode operation for two-dimensional growth, especially for polymers, while voxelated methodologies dominate three-dimensional manufacture.

As automation advances, fully automated and robotic SECCM and related MCED systems are within reach, which will lead to a new generation of high-throughput, data-driven fabrication protocols and real-time adaptive methods. Paired with combinatorial approaches and real-time machine-learning optimization, this evolution stands to accelerate material discovery and deepen the understanding of localized deposition processes at every length scale. From complex multidimensional structure fabrication to nanoscale mechanistic studies, SECCM is a promising and versatile technique for material synthesis and deposition, firmly situated within the MCED landscape.

## 8 Conclusions

Over the past decade, SECCM has evolved from a niche electrochemical imaging technique into a versatile multifaceted platform with applications that extend to localized fabrication at the micro- and nanoscales. This Perspective has highlighted the growing role of SECCM and meniscus-guided techniques in bottom-up fabrication, where spatial confinement of electrochemical reactions enables precise control over crystal nucleation and growth, surface modification, and polymerization.

By tuning parameters such as the applied potential, meniscus size, and electrolyte composition, SECCM offers a high degree of tunability over the structure, chemical composition, and morphology of resulting materials. Furthermore, the ability of SECCM to approach ultrahigh resolution has enabled the capture of stochastic single-nucleation events in tiny droplet cells, reporting phenomena that are inaccessible to macroscale electrochemical methods. Dual-barrel configurations of SECCM offer material deposition on a wide range of surfaces—conducting and insulating, flat and rough, and with uniform or heterogeneous properties. In addition, dual-barrel SECCM permits multimaterial deposition with reagent mixing that is local and at nanoscale dimensions. These capabilities offer significant advantages for applications requiring precise surface engineering and site-specific deposition.

Recent advances in multimodal integration, with microscopy, spectroscopy, and optical feedback, are expanding the use of SECCM to direct electrochemical transformations which will begin to simplify and improve the characterization of material synthesis and deposition via SECCM. In parallel, the adoption of automation and artificial-intelligence-assisted workflows is streamlining data interpretation and enabling adaptive control during scanning and deposition procedures.

As SECCM continues to evolve, it is important that it be recognized as a key contributor to the emerging MCED landscape. At the same time, both communities stand to benefit from embracing the other perspective, allowing conceptual foundations to be shared and inviting opportunities for cross-disciplinary innovation. As technical challenges continue to be addressed and automation becomes more widespread, SECCM is well positioned to contribute meaningfully to the MCED field, aiding in the development of next-generation materials for energy, electronics, biotechnology, and beyond.

## Acronyms and Abbreviations

2D: two-dimensional
3D: three-dimensional
ADF-STEM: annular dark field-scanning transmission electron microscopy
AFM: atomic force microscopy
AQDS: anthraquinone-2,6-disulfonate
EDS: energy-dispersive X-ray spectroscopy
FIB: focused ion beam
FluidFM: fluidic force microscopy
FRP: free-radical polymerization
GC: glassy carbon
HOPG: highly orientated pyrolytic graphite
IRM: interference reflection microscopy
ITO: Indium tin oxide
MCED: meniscus-confined electrochemical deposition
nano-FTIR: nanoscale-Fourier transform infrared spectroscopy
NPs: nanoparticles
PEDOT: poly­(3,4-ethylenedioxythiophene)
PEDOT/PSS: poly­(3,4-ethylenedioxythiophene)/poly­(styrenesulfonate)
PMMA/PPy: poly­(methyl methacrylate)/polypyrrole
QRCE: quasi-reference counter electrode
RDRP: reversible-deactivation radical polymerization
SAMs: self-assembled monolayers
SECCM: scanning electrochemical cell microscopy
SEM: scanning electron microscopy
SICM: scanning ion conductance microscopy
SPM: scanning probe microscopy
s-SNOM: scattering-type scanning near-field optical microscopy
STM: scanning tunneling microscopy
TEM: transmission electron microscopy
TERS: tip-enhanced Raman spectroscopy
XPS: X-ray photoelectron spectroscopy
